# *Rickettsia lusitaniae* in Ornithodoros Porcinus Ticks, Zambia

**DOI:** 10.3390/pathogens10101306

**Published:** 2021-10-12

**Authors:** Simbarashe Chitanga, Herman M. Chambaro, Lavel C. Moonga, Kyoko Hayashida, Junya Yamagishi, Walter Muleya, Katendi Changula, Benjamin Mubemba, Manyando Simbotwe, David Squarre, Paul Fandamu, King S. Nalubamba, Yongjin Qiu, Sawa Hirofumi, Edgar Simulundu

**Affiliations:** 1 Department of Paraclinical Studies, Faculty of Health Sciences and Veterinary Medicine, School of Veterinary Medicine, University of Namibia, Windhoek Private Bag 13301, Namibia; 2 Department of Biomedical Sciences, School of Health Sciences, University of Zambia, Lusaka P.O. Box 50110, Zambia; 3 School of Life Sciences, College of Agriculture, Engineering and Sciences, University of KwaZulu—Natal, Private Bag X54001, Durban 4000, South Africa; 4 International Institute for Zoonosis Control, Hokkaido University, N 20 W10, Kita-ku, Sapporo 001-0020, Japan; herman@czc.hokudai.ac.jp (H.M.C.); lavelmwanga@gmail.com (L.C.M.); kyouko-h@czc.hokudai.ac.jp (K.H.); junya@czc.hokudai.ac.jp (J.Y.); davidsquarre@yahoo.co.uk (D.S.); yongjin_qiu@czc.hokudai.ac.jp (Y.Q.); h-sawa@czc.hokudai.ac.jp (S.H.); 5 Central Veterinary Research Institute (CVRI), Ministry of Fisheries and Livestock, Lusaka P.O. Box 33980, Zambia; pfandamu@gmail.com; 6 Department of Biomedical Sciences, School of Veterinary Medicine, University of Zambia, Lusaka P.O. Box 32379, Zambia; waltermuleya@gmail.com; 7 Department of Paraclinical Studies, School of Veterinary Medicine, University of Zambia, Lusaka P.O. Box 32379, Zambia; katendi.changula@gmail.com; 8 Department of Biomedical Sciences, Michael Chilufya Sata School of Medicine, Copperbelt University, Kitwe P.O. Box 21692, Zambia; mubembab85@yahoo.co.uk; 9 Department of Wildlife Sciences, School of Natural Resources, Copperbelt University, Kitwe P.O. Box 21692, Zambia; 10 Department of Disease Control, School of Veterinary Medicine, University of Zambia, Lusaka P.O. Box 32379, Zambia; simbotwemanyando@gmail.com; 11 Wildlife Diseases Unit, Ministry of Fisheries and Livestock, Lusaka P.O. Box 50060, Zambia; 12 Department of Clinical Studies, School of Veterinary Medicine, University of Zambia, Lusaka P.O. Box 32379, Zambia; king.nalubamba@unza.zm; 13 International Collaboration Unit, International Institute for Zoonosis Control, Hokkaido University, N 20 W10, Kita-ku, Sapporo 001-0020, Japan; 14 Global Virus Network, 725 W Lombard St., Baltimore, MD 21201, USA; 15 Macha Research Trust, Choma P.O. Box 630166, Zambia

**Keywords:** Rickettsiae, *Rickettsia lusitaniae*, *Ornithodoros porcinus*, Argasid, Zambia

## Abstract

Rickettsial pathogens are amongst the emerging and re-emerging vector-borne zoonoses of public health importance. Though traditionally considered to be transmitted by ixodid ticks, the role of argasid ticks as vectors of these pathogens is increasingly being recognized. While bat-feeding (*Ornithodoros faini*) and chicken-feeding (*Argas walkerae*) argasid ticks have been shown to harbor *Rickettsia* pathogens in Zambia, there are currently no reports of *Rickettsia* infection in southern Africa from warthog-feeding (*Phacochoerus africanus*) soft ticks, particularly *Ornithodoros moubata* and *Ornithodoros porcinus*. Our study sought to expand on the existing knowledge on the role of soft ticks in the epidemiology of *Rickettsia* species through screening for *Rickettsia* pathogens in warthog burrow-dwelling soft ticks from two national parks in Zambia. The tick species from which *Rickettsia* were detected in this study were identified as *Ornithodoros porcinus*, and an overall minimal *Rickettsia* infection rate of 19.8% (32/162) was observed. All of the sequenced *Rickettsia* were identified as *Rickettsia lusitaniae* based on nucleotide sequence similarity and phylogenetic analysis of the citrate synthase (*gltA*) and 17kDa common antigen (*htrA*) genes. Utilizing all of the *gltA* (*n* = 10) and *htrA* (*n* = 12) nucleotide sequences obtained in this study, BLAST analysis showed 100% nucleotide similarity to *Rickettsia lusitaniae*. Phylogenetic analysis revealed that all of the Zambian *gltA* and *htrA* gene sequences could be grouped with those of *Rickettsia lusitaniae* obtained in various parts of the world. Our data suggest that *Rickettsia lusitaniae* has a wider geographic and vector range, enhancing to our understanding of *Rickettsia lusitaniae* epidemiology in sub-Saharan Africa.

## 1. Introduction

The *Rickettsia* genus is composed of pathogens considered to be amongst the emerging zoonotic vector-borne pathogens [1,2] and can be divided into three primary groups based on genotype: the ancestral, typhus and spotted fever group (SFG) [3]. The common vectors for this genus include ticks, fleas, mites and lice [4]. Whilst the role of hard ticks (Family Ixodidae) as important vectors and reservoirs of *Rickettsia* species is well established [4,5,6], recent evidence indicates that soft ticks (Family Argasidae) also play a significant role as vectors [7,8,9,10,11,12].

Within the tropical areas of Africa, 10 Argasidae species have been defined, with *Ornithodoros moubata* complex (vectors of African swine fever and human relapsing fever), *Ornithodoros porcinus* (vectors of African swine fever) and *Ornithodoros savignyi* (cause of sand tampan toxicosis) being considered to have the greatest veterinary and public health significance [13]. In Africa, the role of soft ticks as vectors of zoonotic pathogens is very well established in the transmission of tick-borne relapsing fever causative agents (*Ornithodoros moubata* complex—*Borrelia duttonii*, *Ornithodoros sonrai*—*Borrelia crocidurae* and *Ornithodoros erraticus*—*Borrelia hispanica*) [14]. However, investigations into the role of soft ticks in the transmission of *Rickettsia* species have been limited, but interest in this line of research is currently gaining momentum.

The circulation of *Rickettsia* species in African soft ticks has been reported in the genera *Ornithodoros*, *Argas* and *Carios,* with the reported Rickettsia including *R. hoogstraalii*, *R. lusitaniae*, *Candidatus* Rickettsia africaseptentrionalis, *Candidatus* R. mauretanica, as well as some unidentified *Rickettsia* species, with these reports emerging from Morocco, Algeria, Ethiopia, Tunisia, Namibia, South Africa and Zambia [7,8,9,10,11,12]. The soft ticks analyzed across Africa have been collected from bat caves, rodent burrows, bird nests and crevices in human and livestock dwellings.

In Zambia, Qiu et al. [10] reported the presence of *R. hoogstraalii* and *R. lusitaniae* in *Argas walkerae* and *Ornithodoros faini* ticks, respectively, with *R. lusitaniae* being reported for the first time in Africa. *R. lusitaniae* is a newly emergent *Rickettsia* species whose pathogenicity to humans, geographical spread and vector and/or host range is yet to be fully elucidated. Considering the reports of *Rickettsia* species in soft ticks in Zambia [10], our study sought to screen for these pathogens in ticks collected from warthog burrows in selected national parks of Zambia, expanding our knowledge on their role in the epidemiology of *Rickettsia* pathogens in the country.

## 2. Results

### 2.1. Tick Sampling and Identification

**Figure 1:**
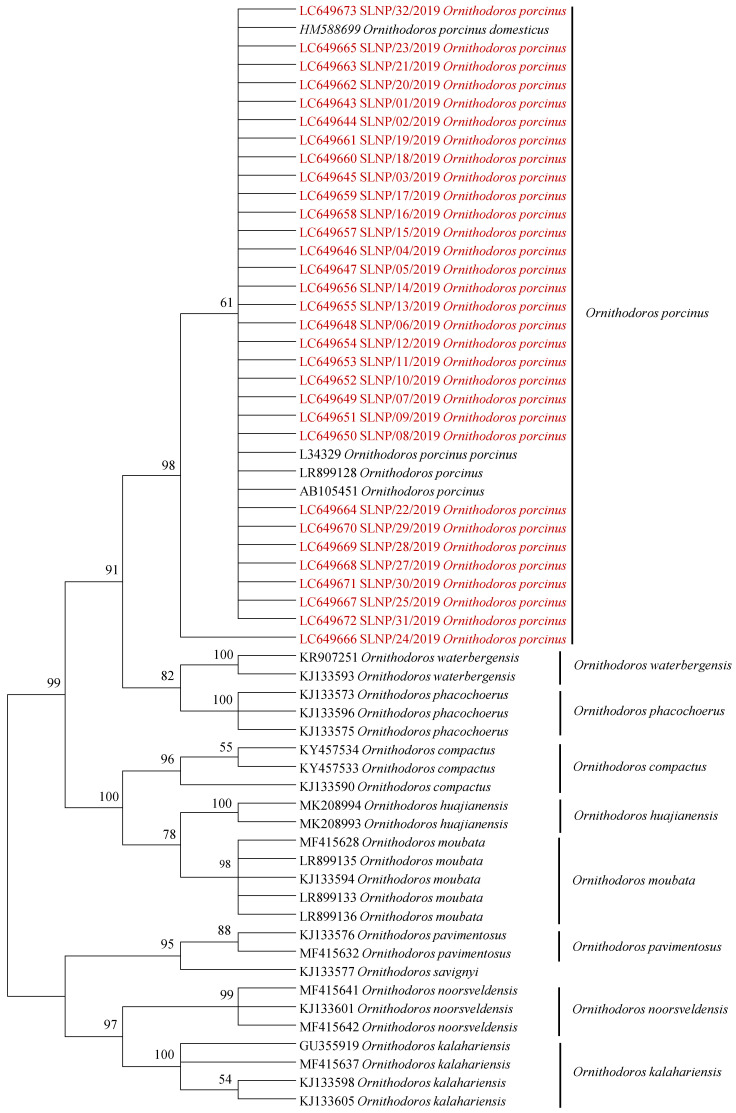
Phylogenetic analysis of the mitochondrial *16S rRNA* gene of soft ticks collected in South Luangwa National Park in Zambia. The phylogenetic tree was generated using the maximum likelihood method. The analysis involved 60 nucleotide sequences with a total of 431 positions in the final dataset. The reference sequences included in the analysis are shown with their GenBank accession numbers and species names, while sequences obtained in this study are in red text. Bootstrap values ≥50% are shown at branch nodes.

A total of 162 ticks, comprising 75 from Kafue National Park (KNP) and 87 from South Luangwa National Park (SLNP), were analyzed in this study. All the ticks were morphologically identified as *Ornithodoros* species and were subsequently pooled into a total of 58 pools (KNP—24, SLNP—34). Further confirmation of the species’ identity was obtained by phylogenetic analysis of the mitochondrial *16S rRNA* gene sequences in those pools that tested positive for *Rickettsia* species. Phylogenetic analysis revealed that the 31 mitochondrial *16S rRNA* gene sequences obtained in this study clustered within the *O. porcinus* complex group (Figure 1).

### 2.2. Rickettsia Screening and Identification

On initial screening for *Rickettsia* using the loop-mediated isothermal amplification (LAMP) technique, an overall minimum infection rate (MIR) of 19.8% (32/162) was observed. When separated by area of sampling, all of the positive pools were from SLNP, while no *Rickettsia* genome was detected in ticks from KNP. The MIR for SNLP was determined as 36.8% (32/87). On statistical analysis, the overall pooled prevalence was determined as 23% [95% CI (16.4–31.8)], with the pooled prevalence for SLNP determined as 61.1% [95% CI (41.8–80.7)].

The sequences obtained from the positive pools, based on the *gltA* gene, showed 100% identity to the *R. lusitaniae* strain ZS13 that had been detected in the heart of the common pipistrelle (*Pipistrellus pipistrellus*) in China (GenBank accession: MN388795). Further sequence comparison based on the *htrA* gene showed 100% nucleotide sequence identity to the *R. lusitaniae* strain OnF11 detected in *O. faini* ticks in Zambia (GenBank accession: LC558319) [10].

**Figure 2:**
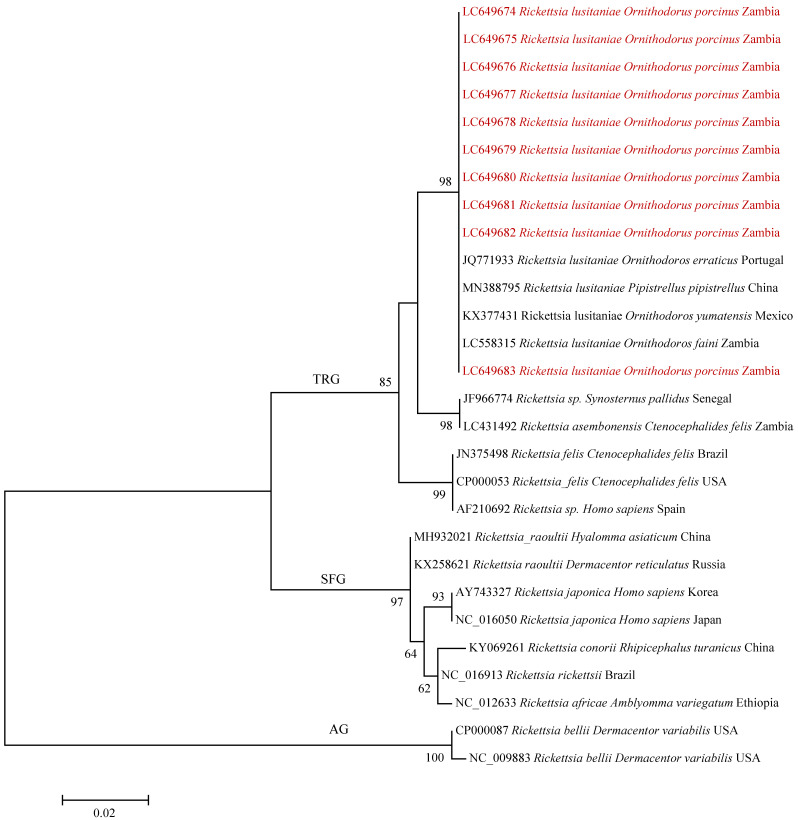
Phylogenetic tree of *Rickettsia* spp. based on *gltA* gene sequences and those detected from *O. porcinus* ticks collected in Zambia (red text). The genetic tree was generated using the maximum likelihood method. The analysis involved 28 nucleotide sequences with a total of 320 positions in the final dataset. The *Rickettsia* spp. are divided into transitional group (TRG), spotted fever group (SFG) and ancestral group (AG). Bootstrap values ≥ 50% are shown at branch nodes. The scale bar shows the number of substitutions per site.

On phylogenetic analysis of the *gltA* gene, the samples in our study clustered with *Rickettsia lusitaniae* were detected in various parts of the world (Figure 2).

**Figure 3:**
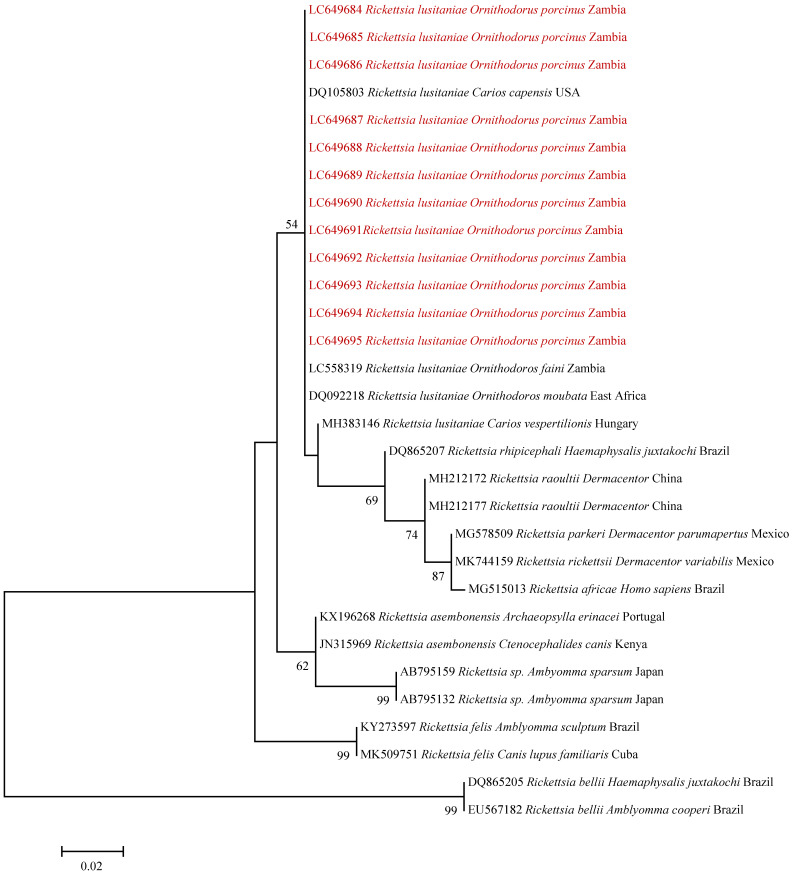
Phylogenetic tree of *Rickettsia* spp. based on *htrA* gene sequences. The maximum likelihood method was used to produce the phylogenetic tree. The analysis involved 30 nucleotide sequences and a total of 233 positions in the final dataset. The sequences detected in *O. porcinus* collected from Zambia are shown in red text. Reference sequences are shown with their accession numbers, *Rickettsia* species, host and country of origin. Bootstrap values ≥ 50% are shown at branch nodes. The scale bar shows the number of substitutions per site.

A similar clustering pattern was observed upon phylogenetic analysis of the *htrA* gene (Figure 3).

## 3. Discussion

In this study, we sought to investigate the presence of *Rickettsia* species in argasid ticks collected from warthog burrows in two national parks in Zambia. To the best of our knowledge, this is the second study that has sought to examine the role of soft ticks in the epidemiology of *Rickettsia* species in the country. The previous study screened for *Rickettsia* and *Anaplasma* species in ticks collected from warthog burrows (*O. moubata*), chicken coops (*Ar. walkerae*) and a bat cave (*O. faini*), with *Rickettsia* species being detected in both *Ar. walkerae* (*R. hoogstraalii*) and *O. faini* (*R. lusitaniae*) species [10]. As such, our study adds to the growing body of knowledge concerning the presence and distribution of *Rickettsia lusitaniae* in argasid ticks, with *O. porcinus* now being added as potential vectors of this group of pathogens. *Rickettsia* species have previously been reported in *Argas* [8,9], *Ornithodoros* [7,11] and *Carios* [11] tick species, which are associated with birds, bats and rodents across the African continent. Our study adds warthogs to the list of possible hosts of soft-tick-associated various *Rickettsia* species, further improving our understanding of the circulation of *Rickettsia* species and their potential vectors in Zambia [10,15,16,17,18].

The observed prevalence in our study was higher than that observed by Qiu et al. [10] [23% vs. 10%]. Whilst the difference in study design (pooling vs. single samples) cannot be discounted as a cause of the observed differences in prevalence, it is also possible that a difference in the tick species sampled, a difference in sampling locations and the hosts on which the ticks were feeding, could also have contributed to the observed difference, especially as these are known to influence the tick microbiome [19,20,21,22,23]. Geographic variation could also explain the absence of *Rickettsia* infection in ticks collected from KNP. It has been suggested that differences in microbial composition of soil can influence the tick microbiome [21], resulting in a difference in the microbial composition in ticks collected from different geographical locations.

The present study is the first to report the presence of bacteria in *O. porcinus* ticks, presenting an expanded geographic range of the bacteria in the country. Our study also reports the presence of bacteria in the eastern part of the country, adding to the findings that Lusaka previously reported [10]. In contrast, the study conducted by Qiu et al. [10] did not detect *R. lusitaniae* in *O. moubata* collected from warthog burrows. The *O. porcinus* in our study was shown to carry this pathogen, thus indicating that warthogs could also play a role in the epidemiology of the pathogen, in addition to the bats previously reported. We also observed a very high infection rate of *R. lusitaniae* in the screened samples. This observation was not surprising considering the fact that *Rickettsia* species are transovarially and transstadially transmitted in ticks [24].

## 4. Materials and Methods

### 4.1. Study Sites

The study was conducted in KNP and SLNP. The KNP, located in south-central Zambia, is the biggest National Park in the country and is approximately 22,400 km^2^ in size. The vegetation is mainly Miombo woodland, Kalahari woodland and swamps. In contrast, the SLNP, located in the Luangwa Valley, eastern Zambia, is roughly 9050 km^2^ in size. The vegetation in SLNP is a combination of Mopane and Miombo woodlands with mountainous Muchinga escarpment. Soft ticks were collected from warthog (*Phacochoerus africanus*) burrows and culverts in the two National Parks as previously described [25,26]. The loose soil and litter were manually collected using a shovel and placed on black polythene bags, after which they were exposed to sunlight to elicit tick movement. The ticks were collected using entomological forceps and kept alive in tick bottles until identification using morphological and molecular techniques [27,28,29]. From each infested burrow, 2–3 adult ticks were picked at random and pooled. DNA was extracted from the pooled ticks using the DNeasy^®^ Blood and Tissue Kit (Qiagen, Hilden, Germany), according to the manufacturer’s guidelines.

### 4.2. Molecular Identification of Soft Ticks

Soft tick pools from SLNP that tested positive for *Rickettsia* were analyzed further using primers 16S+1 (5′-CTGCTCAATGATTTTTTAAATTGCTGTGG-3′) and 16S-1 (5′-CCGGTCTGAACTCAGATCAAGT-3′) to target the mitochondrial 16S *rRNA* gene [27]. The PCR was performed using Ampdirect plus buffer (Shimadzu, Tokyo, Japan) with BioTaq Polymerase (Bioline, London, UK) as described by the manufacturers. The amplification reaction was conducted at one-step enzyme activation at 95 °C for 5 mins and 35 cycles of 94 °C DNA denaturation—1 min, 54 °C annealing—35 s and 72 °C extensions—1.5 min with a final extension at 72 °C—7 min. Amplicons of the expected size were purified from PCR products using AMPure XP beads (Beckman Coulter, High Wycombe, UK) and sequenced directly using the BigDye terminator cycle sequencing ready reaction kit version 3.1 (Applied Biosystems) on a 3500 Genetic Analyzer (Applied Biosystems, Foster City, CA, USA). The obtained sequences were assembled and edited using GENETYX ATGC software version 7.5.1 (GENETYX Corporation, Tokyo, Japan). Sequences were deposited in the DDBJ GenBank under the accession numbers LC649643–LC649673.

Phylogenetic analysis was used to confirm the tick species. Evolutionary history was inferred in MEGA6 using the maximum likelihood method based on the Hasegawa–Kishino–Yano model. Model selection was conducted using MEGA6. Discrete Gamma distribution was used to model the evolutionary rate differences between sites (5 categories (+G, parameter = 0.4299)). The analysis involved 60 nucleotide sequences, and all positions containing gaps and missing data were eliminated. The bootstrap method (with 1000 replicates) was used to assess the phylogenetic tree topological reliability.

### 4.3. Molecular Screening and Identification of Rickettsia Species

Tick genomic DNA samples were subjected to loop-mediated isothermal amplification (LAMP) for detection of rickettsial DNA by targeting the 17-kDa fragment, using the following primers: Rr17F3 (5’-TGTTACAAGCCTGTAACGG-3’), Rr17B3 (5′-TCCTGTTCATCCATACCTG-3′), Rr17FIP (5′-GAGAACCAAGTAATGCGCCGGGCGGTATGAATAAACAAGG-3′), Rr17BIP (5′-AATTCGGTAAGGGCAAAGGACCACCGATTTGTCCACCAA-3′), Rr17LoopF (5′-F1-X-CCGCCAAGAAGTGTTCCTGTA-3′) and Rr17LoopB (5′-Biotin-AGCTTGTTGGAGTAGGTGTAGGTG-3′) [30]. The LAMP reactions were performed at 62 °C for 60 min and monitored using the Rotor-Gene 3000 thermocycler (Corbett Research, Sydney, Australia), after which melting temperature analysis was performed. For the purpose of sequencing using the Sanger method, samples that were positive on the LAMP analysis were randomly selected for amplification using conventional PCR targeting of the 581-bp fragment of the citrate synthase gene [31] or the 550-bp fragment of the 17-kDa gene [32]. The PCR was performed as described under amplification of soft tick DNA.

PCR product purification, sequencing, assembly and editing was conducted as described under molecular identification of ticks. Sequences were deposited in the DDBJ GenBank under the accession numbers LC649674–LC649695. For phylogenetic analysis, maximum likelihood trees for the *gltA* and *htrA* gene sequences were generated in MEGA6 using the Tamura 3-parameter and Kimura 2-parameter evolutionary models as determined in MEGA6, respectively. Topological support was assessed using the bootstrap method with 1000 replicates.

### 4.4. Statistical Analysis 

The results from the LAMP analysis were used to determine the MIR, which was calculated as the proportion of tick pools that showed amplification of the target gene, out of the total number of ticks tested, multiplied by 100 [33]. Pooled prevalence estimates for perfect tests with exact confidence limits were calculated using EpiTools epidemiological calculators [34], assuming 100% test sensitivity and specificity for a fixed pool size. Exact confidence (95% CI) limits were calculated based on binomial theory [35]. 

## 5. Conclusions

We report the detection of *R. lusitaniae* in *O. porcinus* ticks in Zambia. Our study adds to the growing knowledge of this newly emergent *Rickettsia* species as well as tick species involved in the epidemiology of *Rickettsia* species in Zambia, with previous reports concerning hard ticks indicating the circulation of a number of *Rickettsia* species, some of which are of known zoonotic potential. Considering the apparent widespread presence of *Rickettsia* species in hard and soft ticks in Zambia, there is a need for further studies in order to explore various tick–host associations and to gain a more comprehensive understanding of the epidemiology of these pathogens in the country. 

## Data Availability

All sequences generated from this study have been uploaded on the public database, DDBJ GenBank under accession numbers LC649643–LC649695.
